# Stigma against People Living with HIV/AIDS in China: Does the Route of Infection Matter?

**DOI:** 10.1371/journal.pone.0151078

**Published:** 2016-03-16

**Authors:** Chen Zhang, Xiaoming Li, Yu Liu, Shan Qiao, Liying Zhang, Yuejiao Zhou, Zhenzhu Tang, Zhiyong Shen, Yi Chen

**Affiliations:** 1 Divison of Epidemiology, Vanderbilt University, Nashville, Tennessee, United State of America; 2 Department of Health Promotion, Education, and Behavior, Arnold School of Public Health, University of South Carolina, Columbia, South Carolina, United State of America; 3 Department of Pediatrics, Wayne State University, Detroit, Michigan, United State of America; 4 Department of HIV/STD Prevention, Guangxi CDC, Nanning, Guangxi, China; National Center for AIDS/STD Control and Prevention, China CDC, CHINA

## Abstract

In the current study, we tested the hypothesis that people who contracted HIV from “blameless” routes (e.g., blood transfusion, sex with stable partners) are less stigmatized compared to people who contracted HIV from “blamable” routes (e.g., injection drug use, sex with sex workers). A cross-sectional study was conducted among 2,987 participants in Guangxi province, China, between 2012 and 2013. We employed both explanatory and predictive modeling strategy by using multivariate linear regression models. In the explanatory models, we assessed the association between routes of infection and three types of stigma (perceived, internalized, and enacted). From identified routes of infection that significantly contributed to higher stigma, we employed predictive modeling to explore predictors for the specific type of stigma. Multiple-imputation was employed for sensitivity analyses. Of the total sample, 63% were male and the average age was 42.9 years (ranged between 18 and 88). Multivariate regression models revealed that contraction from commercial sex increased the perceived (β = 0.46, 95%CI = 0.02, 0.90) and internalized stigma (β = 0.60, 95%CI = 0.09, 1.10), while injecting drug use increased the perceived (β = 0.65, 95%CI = 0.07, 1.22) and enacted stigma (β = 0.09, 95%CI = 0.02, 0.16) after controlling for confounders. Among PLWHA who were infected via commercial sex partners, social support was negatively associated with perceived (β = -0.47, 95%CI = -0.79, -0.14) and internalized stigma (β = -0.80, 95%CI = -1.24, -0.35). Among PLWHA who were infected via injecting drugs, no adherence to antiretroviral treatment (β = 0.41, 95%CI = 0.01, 0.82) was positively associated with perceived stigma, and disclosure of serostatus to others was negatively associated with enacted stigma (β = -0.20, 95%CI = -0.34, -0.05). Knowledge of the association between routes of infection and stigma can guide health professionals and policy makers to develop tailored intervention strategies to mitigate the effects of stigma and enhance HIV care utilization among PLWHA in China.

## Introduction

With a reported 501,000 people living with HIV/AIDS (PLWHA) by the year of 2014 (population HIV prevalence: 0.058%[0.046%-0.070%]) [1], China remained a low HIV epidemic country. The low HIV prevalence may lead to intensified stigma against PLWHA due to less tolerance of the disease and greater fear of HIV compared to high-prevalence settings[2–5]. For instance, Cao et al. (2009) indicated that PLWHA felt less stigmatized when living in HIV-endemic areas compared to peers living in low-prevalence regions[3,6]. Meanwhile, a study conducted in a rural area of China revealed that almost half of the participants blamed PLWHA for their disease, and 73% of them considered having HIV was shameful[5].

Previous studies suggested that PLWHA mainly reported three types of stigma embedded within their living contexts, including perceived stigma, internalized stigma and enacted stigma[6–9]. Perceived stigma is an individual’s subjective awareness of discriminatory and prejudicial attitudes from people around[9]. Internalized stigma imposes individuals with unpleasant beliefs towards themselves after incorporating negative views from others[9]. Enacted stigma is defined as an individual’s real experience from external discrimination after disclosure of their HIV-positive status[9]. These three types of stigma may be attributable to different risk factors[10–12] and lead to various sequelae, including severe physical, emotional and financial burdens among PLWHA[2,13–16].

An ecological framework can better illustrate the dynamics of stigma against PLWHA in China[17–19]. At individual level, people with an older age[20], more years of education[21,22], no adherence to ART[23,24], worse physical condition[22,25], poor HIV-related knowledge[20], and poor mental health status[2,11,26] were associated with a higher degree of stigma (either perceived o enacted). At interpersonal level, better social support from family members or friends can mitigate stigmatizing experience among PLWHA[20,27]. At institutional level, environmental factors embedded in working and healthcare settings (e.g. attitudes and behaviors of coworkers or healthcare providers) may affect the stigmatizing experience encountered by PLWHA[16,28,29]. Community-level characteristics may also explain a significant part of stigma that PLWHA have experienced [2,30,31]. For instance, a study conducted among 5,658 respondents from 66 communities in China revealed that at least one-third variance of stigma was attributable to community-level characteristics after accounting for individual level characteristics[30]. At the social level, HIV-phobia and institutional discrimination against PLWHA are common in China[32–34], which further exacerbates stigma against PLWHA.

Although factors embedded within the living context of PLWHA can partially contribute to stigma they encountered, published studies also indicated that the routes of infection may also impact the person’s stigmatized experience[6,35,36]. A qualitative study conducted in Malaysia indicated that HIV infection by injecting drugs or sexual promiscuity was deemed as a punishment for their lifestyle improprieties or committed crimes, while people infected by medical accidents (e.g., blood transfusion) were considered as “innocent” or “blameless” victims[27]. However, very few studies quantitatively assessed magnitudes of associations between routes of infection and different types of stigma encountered by PLWHA, and no such studies have ever been conducted in Chinese setting. To address the research gap, we conducted the current study to test the hypothesis that routes of infection may be associated with different magnitudes and directionality of perceived, internalized and or enacted stigma among PLWHA in China.

## Materials and Methods

### Study design

The current study was conducted from 2012 to 2013 in Guangxi Zhuang autonomous region (Guangxi) located in the Southwest of China. Details of the study setting and design have been described elsewhere[37,38]. In short, we used a pre-established sampling scheme to select top 12 sites with largest cumulative HIV cases from Guangxi’s 17 cities and 75 counties. Approximately 10% HIV cases were randomly selected from a sampling pool with 29,606 HIV/AIDS cases in the 12 sites. With an approximate 10% refusal rate, a total of 3,002 PLWHA were recruited. Two thousand nine hundred and eighty seven of them (99.5%) completed the survey and were included in the current data analysis. The Institutional Review Boards at Wayne State University in the United States and Guangxi Center for Disease Control and Prevention in China reviewed and approved the research protocol.

### Measurements

#### Background information

Participants were asked to provide demographic information regarding their gender (male vs. female), age (years), years of schooling (years), ethnicity (Han, Zhuang, or others), religion (e.g., no-religious, Buddhism, and others), current marital status (e.g., never vs. ever married), place of original residence (urban vs. rural), and total number of children in the household.

Financial situation was measured by monthly household income in Chinese Yuan (6.2 Yuan = 1 USD at the time of the survey: <999, 1000–1999, 2000–2999, ≥3000), employment status (not work at all, part-time, and full-time), and balance between income and expenditure (not enough, barely enough, and enough).

Participant’s physical condition was evaluated based on their most recently available CD4 counts (≤250 cells/ml, 251–500 cells/ml, and >500 cells/ml), viral loads (1–49 copies/ml, 50–1000 copies/ml, and >1000 copies/ml), co-infection with other sexually transmitted diseases (STDs) (yes vs. no), self-rated physical condition (excellent, very good, good, average, poor), degree of pain in the past month (not at all, very slight, average, severe, and very severe), and degree of their daily lives affected by HIV (not at all, a little bit, average, fairly, and significantly). The physical activity capacity in the past month was measured by six items asking participants to what extends (e.g., affected a lot, affected a little, not affected at all) the disease affected their daily activities (e.g., showering, walking for a mile, climbing stairs, and running). We summed the responses to the six items with a higher score indicating a better physical activity capacity. The Cronbach’s α of the physical activity scale was 0.92 in the current study.

Psychological health was measured by a few tailored scales including self-esteem (α = 0.94), resilience (α = 0.96), coping skills (α = 0.93), social support (α = 0.98), depression (α = 0.76), and anxiety (α = 0.91). These assessment scales have been validated in previous studies conducted in Chinese settings [37,39].

We also collected information regarding other health risk behaviors including history of drug use (yes vs. no) or alcohol drinking (yes vs. no) in the past six months. In addition, we asked participants if they had disclosed their serostatus to other people (yes vs. no), and perceived attitudes from medical staffs who provided services (everyone is friendly, majority are friendly, half are friendly, less than half are friendly, and very few are friendly).

#### HIV stigma

Three types of stigma including perceive, internalized and enacted stigma were measured by an existing validated HIV stigma scale [37,38,40]. Each stigma item was measured by a Likert-type scale (e.g., strongly disagree, disagree, agree, strongly agree) with a higher score indicating a greater agreement with the statement. Perceived stigma was measured by six items related to awareness of societal norms and prejudicial actions towards PLWHA (e.g., “most people consider PLWHA filthy”). The perceived stigma scale scored from 6 to 24 with a Cronbach’s α = 0.905. Internalized stigma was evaluated by eight items related to their negative feelings about themselves for contracting HIV (e.g., I feel guilty because I have HIV). The internalized stigma scale scored from 7 to 32 with a Cronbach’s α = 0.915. Enacted stigma was measured by two items regarding discriminatory experience participants had encountered (e.g., I will lose my job if my serostatus is known by others). In the current study, the enacted stigma scale scored from 1 to 6 with a Cronbach’s α of 0.627.

#### Routes of infection

Participants were asked to identify one of the following routes of their HIV infection: sex with stable partners, sex with casual partners, sex with commercial partners, blood product contamination, injecting drug use, others, or unknown.

### Statistical analysis

One-way ANOVA was conducted to assess significance of differences among demographic, psychological, physical, contextual factors by routes of infection among 2,987 PLWHA in the current analysis.

We used individual explanatory models to assess the associations between routes of infection (exposure) and each of the three types of stigma (outcome) using multivariate linear regression. As all outcomes (e.g., perceived, internalized and enacted stigma) were continuous variables, we employed the multivariate linear regression models and reported corresponding beta-coefficients and their 95% confidence interval (CI) for each identified predictors. For each specific model, we used *a priori* evidence and directed acyclic graphs (DAGs) to identify potential confounders to fit into the multivariable model [41]. Then, we employed the 10% change-in-estimate to retain a minimum sufficient set of confounders in each final model [42]. To handle the missing data issue, we conducted multiple imputation, and both results from complete-case and imputed analyses were reported.

After identifying how each specific infection route affected different types of stigma, we further conducted a subgroup analysis to explore predictors for the specific infection route which was significantly associated with any of the three types of stigma among PLWHA. For instance, if we found infection via commercial sex was associated with perceived stigma, we further assessed personal, psychological and contextual predictors of perceived stigma among the participants who were specifically infected via commercial sex. As each specific type of stigma was continuous variables, we employed the multivariate linear regression models and reported corresponding beta-coefficients and their 95% CI for each identified predictor. To follow the procedure of establishing predictive models, multivariate linear regression with backward selection and LRT for global test (Chi-square test for model with all the terms vs. a model with only the intercept) were employed [43]. We used the *P*-value of 0.20 as the cut-off point for both the global LRT and the stepwise regression tests. For models with more than 15% of missing values, we also employed the multiple imputation strategy in the sensitivity analyses [44]. All analyses were conducted using the STATA^®^ package (Version 12, College Station, TX).

## Results

### General description of the characteristics of PLWHA

A total of 2,987 participants were included in the data analysis. The most frequently reported infection route was having sex with stable partners (28.5%), followed by having sex with commercial partners (21.5%), injecting drug use (15.8%), having sex with casual partners (15.7%), using blood products (1.0%), and others (0.8%). In addition, 16.3% of them reported “unknown” about their infection route in the current sample. The mean score of perceived, internalized and enacted stigma encountered by PLWHA was 15.53(SD = 3.53), 18.49(SD = 4.35), and 2.09(SD = 0.45), respectively. People who contacted the virus via injecting drug use had the worst scores on all three types of stigma compared to their peers who were infected via other routes (*P*<0.05; Table 1).

**Table 1:** Demographic, psychological, contextual factors by route of infection.

	Sex with stable partners (n = 852)	Sex with casual partners (n = 468)	Sex with commercial partners (n = 642)	Blood products (n = 30)	Injecting drug use (n = 471)	Others (n = 24)	Unknown (n = 487)	Total (n = 2987)
**Demographics**								
**Gender**								
female	76.76	22.86	11.68	50.00	10.62	54.17	39.40	37.19****
males	23.24	77.14	88.32	50.00	89.38	45.83	60.60	62.81
**Ethnicity**								
Han	67.37	71.58	70.83	46.67	81.28	47.83	68.07	70.72****
Zhuang	28.76	25.85	25.9	50	17.23	47.83	26.91	25.92
Others	3.87	2.56	3.28	3.33	1.49	4.35	5.02	3.35
**Religious**								
Atheist	92.3	89.78	92.2	96.55	95.29	95.83	91.30	92.26
Buddhism or others	7.7	10.22	7.8	3.45	4.71	4.17	8.70	7.74
**Residence**								
Urban	21.86	24.79	13.55	30	17.27	13.04	21.84	19.82****
Rural	78.14	75.21	86.45	70	82.73	86.96	78.16	80.18
**Marital status**								
Never married	30.95	34.65	30.08	13.79	46.48	28.57	30.69	33.53****
Ever married	69.05	65.35	69.92	86.21	53.52	71.43	69.31	66.47
**Age, mean(SD)**	40.67(11.89)	44.03(13.72)	47.84(13.79)	40.73(10.54)	38.26(7.98)	40.54(12.72)	44.13(13.67)	42.94(12.83)****
**Year of school, mean(SD)**	6.98(3.06)	7.50(3.12)	6.63(2.89)	7.28(2.82)	6.84(2.45)	6.91(3.28)	7.02(3.32)	6.97(3.00)****
**Total number of child,mean(SD)**	1.84(1.29)	1.64(1.40)	2.14(1.59)	1.80(1.21)	1.22(1.12)	1.67(1.79)	1.83(1.55)	1.77(1.43)****
**Employment status**								
No work	22.08	29.34	23.13	33.33	35.61	25.00	29.18	26.90****
Part-time	33.06	32.12	34.69	23.33	40.09	20.83	28.17	33.36
Full time	44.86	38.54	42.19	43.33	24.31	54.17	42.66	39.74
**Make ends meet**								
More than Enough	9.18	17.34	14.53	0	3.18	8.33	9.22	10.57****
Barely enough	46.59	35.76	49.53	50	23.14	16.67	34.47	39.58
Not enough	44.24	46.9	35.94	50	73.67	75	56.31	49.85
**Monthly income**								
<1000	51.83	48.59	44.43	60.71	69.08	66.67	54.66	53.14****
1000–1999	32.19	28.42	33.91	28.57	19.62	25	29.35	29.41
2000–2999	11.12	13.23	14.29	7.14	7.89	4.17	9.72	11.29
> = 3000	4.85	9.76	7.38	3.57	3.41	4.17	6.28	6.15
**Psychological conditions**								
***Self-esteem*, *mean(SD)***	3.41(0.70)	3.32(0.77)	3.33(0.68)	3.28(0.80)	3.20(0.73)	3.16(0.83)	3.24(0.71)	3.31(0.72)
***Resilience*, *mean(SD)***	3.24(0.83)	3.26(0.85)	3.22(0.83)	3.54(0.73)	3.06(0.80)	3.28(0.86)	3.10(0.89)	3.19(0.84)
***Coping strategy*, *mean(SD)***	2.53(0.64)	2.56(0.63)	2.55(0.59)	2.47(0.76)	2.64(0.69)	2.48(0.83)	2.38(0.66)	2.53(0.65)***
***Social support*, *mean(SD)***	2.51(0.88)	2.44(0.89)	2.40(0.79)	2.51(1.09)	2.35(0.78)	2.63(1.04)	2.33(0.85)	2.42(0.85)***
***Depression*, *mean(SD)***	7.57(4.59)	7.29(4.83)	7.37(4.46)	7.67(4.79)	9.11(5.39)	8.88(5.99)	7.82(5.24)	7.78(4.89)
***Anxiety*, *mean(SD)***	30.17(8.35)	30.75(8.76)	30.17(7.94)	32.77(10.16)	33.43(10.53)	35.04(11.54)	31.61(8.55)	31.08(8.87)
**Physical conditions**								
***CD4 groups(counts/ml)***								
***< = 250***	30.92	38.12	40.29	33.33	34.63	37.5	39.67	36.18*
***250–500***	47.46	45.74	41.59	43.33	42.43	45.83	41.34	44.08
***>5000***	21.62	16.14	18.12	23.33	22.94	16.67	19	19.75
***Viral load groups(copies/ml)***								
***1–49***	76.09	77.99	67.85	80.00	71.63	72.73	77.69	74.25****
***50–1000***	17.84	13.13	25.34	15.00	17.79	27.27	9.56	17.53
***>1000***	6.07	8.88	6.81	5.00	10.58	0.00	12.75	8.22
***Physical activity scale***	11.84(2.88)	11.31(3.04)	11.68(2.82)	12.20(2.86)	10.78(3.05)	11.54(3.22)	11.14(3.04)	11.44(2.97)
***Self-rate Physical condition***								
Excellent	4.46	5.98	3.43	6.67	2.34	4.17	2.61	3.85****
very good	17.14	16.67	13.55	13.33	8.09	20.83	13.63	14.27
Good	25.59	23.5	25.7	43.33	17.45	16.67	26.25	24.22
average	42.96	41.88	46.42	33.33	48.51	54.17	46.29	44.96
Poor	9.86	11.97	10.9	3.33	23.62	4.17	11.22	12.70
***Degree of pain***								
not at all	55.53	50.00	51.09	70.00	43.4	65.22	44.38	50.15*
very slight	12.71	13.68	13.13	0	11.91	0	13.45	12.72
Slight	11.06	13.46	14.22	10.00	16.81	8.7	15.46	13.73
average	16.24	17.09	17.03	20.00	20.64	26.09	21.29	18.19
Severe	3.41	4.91	3.75	0.00	6.17	0	4.42	4.26
very severe	1.06	0.85	0.78	0.00	1.06	0	1.00	0.94
***If disease affects daily life***								
not at all	46.75	40.95	45.92	50	35.13	50.00	36.22	42.13****
a little	26.63	25	23.82	13.33	25.22	8.33	27.57	25.42
average	16.57	20.69	19.44	23.33	22.63	20.83	22.13	19.82
Significantly	7.69	9.27	7.68	6.67	12.93	12.5	8.45	8.91
Very significantly	2.37	4.09	3.13	6.67	4.09	8.33	5.63	3.71
***Co-infection***								
Never	89.07	82.13	85.35	78.57	62.63	77.27	82.05	81.75****
Ever	10.93	17.87	14.65	21.43	37.37	22.73	17.95	18.25
**Risky Behaviors**								
***drinking alcohol***								
Never	72.59	53	43.91	56.67	44.89	87.50	61.65	57.12****
Ever	27.41	47	56.09	43.33	55.11	12.50	38.35	42.88
***Drug use***								
Never	94.35	94.84	92.52	90	15.63	83.33	89.11	80.67****
Ever	5.65	5.16	7.48	10	84.37	16.67	10.89	19.33
***Have sex in past 6m***								
Never	41.06	43.68	58.19	26.67	50.96	47.83	48.80	47.92****
Ever	58.94	56.32	41.81	73.33	49.04	52.17	51.20	52.08
**Contextual factors**								
***Disclosure***								
Never	86.96	71.12	78.66	80	80.38	91.67	73.55	79.39****
Ever	13.04	28.88	21.34	20	19.62	8.33	26.45	20.61
***Attitude from medical staff***								
All are good	79.44	75.97	80.34	70	78.13	70.83	77.76	78.44
Majority are good	17.51	19.96	16.22	26.67	16.35	20.83	17.43	17.54
Half are good	1.76	2.58	2.18	3.33	2.76	4.17	3.21	2.41
Less than half are good	0.82	0.64	1.09	0	2.12	4.17	1.00	1.11
Only a few are good	0.47	0.86	0.16	0	0.64	0	0.60	0.50
**Stigma**								
**Perceived stigma, mean(SD)**	15.30(3.46)	14.98(3.71)	15.71(3.35)	15.20(4.77)	16.39(3.17)	15.38(3.55)	15.42(3.81)	15.53(3.53)****
**Internalized stigma, mean(SD)**	18.04(4.09)	18.09(4.54)	18.66(4.22)	19.13(5.88)	19.24(4.13)	18.38(4.76)	18.67(4.74)	18.49(4.35)****
**Enacted stigma, mean(SD)**	2.07(0.37)	2.08(0.43)	2.05(0.29)	2.20(0.66)	2.18(0.64)	2.17(0.82)	2.11(0.51)	2.09(0.45)****

Notes:

*p<0.05,

***p<0.001,

****p<0.0001;

SD: standard deviation

A majority of the participants were males (62.81%). Two-thirds were ever married (66.47%), 70.72% were of Han-ethnicity, and 80.18% were living in rural areas. The mean age of the current sample was 42.94(SD = 12.83) with approximately seven years of formal schooling. One-third of them had full-time job and half of them cannot make ends meet financially. Males were more likely to report infection via sex with casual/commercial partners or injecting drug use, while females were more likely to get infected by having sex with their stable partners (p<0.05; Table 1).

Among all participants, 42.9% of them ever drank alcohol, 19.3% used drugs, and 52.1% had sex in the past six months. About one-fifth disclosed their serostatus to others, and 96% reported experiencing friendly attitudes from a majority of medical professionals. More than one-third of them had CD4 counts less than 250 cells/ml (30.92% for at least 250 cells/ml, 47.46% for 250–500 cells/ml, and 21.62% for more than 500 cells/ml), and 26% of them had viral load higher than 50 copies/ml (76.09% for 1–49 copies/ml, 17.84% for 50–1000 copies/ml, and 6.07% for more than 1000 copies/ml). About half of them self-rated their health condition as “just average” or “poor”; more than 5% reported having a “severe” or “very severe” degree of pain, and more than 10% said the HIV had affected their daily life significantly (8.9%) or very significantly (3.7%). Eighteen percent of them had co-infections with STDs in the current sample.

PLWHA who were infected via injecting drug use usually had the worse physical, emotional, and financial conditions. Specifically, those who reported infection via injecting drug use had the lowest physical activity score (mean = 10.8, SD = 3.1), and more likely to report severely painful experience (24.6%) and significantly impacted daily activities (7.2%) due to the diseases. In addition, they reported highest scores of anxiety (mean = 33.4, SD = 10.5) and depression (mean = 9.1, SD = 5.4), and least likely to make ends meet (*P*<0.05; Table 1).

### Explanatory models

In complete-case analyses, we found injecting drug use increased the likelihood of experiencing perceived (β = 0.65, 95%CI = 0.07, 1.22) and enacted (β = 0.09, 95%CI = 0.02, 0.16) stigma; sex with commercial sex increased the perceived (β = 0.46, 95%CI = 0.02, 0.90) and internalized stigma (β = 0.60, 95%CI = 0.09, 1.11). Results from the multiple imputation indicated the same significant risk factors for each type of stigma as we found with complete-case analyses (Table 2).

**Table 2:** Multivariate analysis between stigma and route of infection (N = 2987).

	Perceived stigma^a^ (β,95%CI)		Internalized stigma^b^ (β,95%CI)		Enacted stigma^c^ (β,95%CI)	
Routes of Infection	Complete Cases (n = 2401)	Multiple Imputation (n = 2879)	Complete Cases (n = 2401)	Multiple Imputation (n = 2879)	Complete Cases (n = 2440)	Multiple Imputation (n = 2906)
*Sex with stable partners*	Ref	Ref	Ref	Ref	Ref	Ref
***Sex with causal partners***	-0.23(-0.68,0.22)	-0.18(-0.60,0.24)	-0.058(-0.58,0.47)	-0.00002(-0.49,0.49)	0.001(-0.06,0.06)	0.02(-0.04,0.07)
***Sex with sex workers***	0.46(0.02,0.90)*	0.48(0.07,0.89)*	0.60(0.09,1.11)*	0.57(0.10,1.04)*	-0.010(-0.069,0.05)	-0.008(-0.06,0.05)
***Blood transfusion***	-.035(-1.71,1.01)	0.09(-1.19,1.36)	1.06(-0.52,2.64)	1.19(-0.27,2.65)	0.15(-0.02,0.33)	0.14(-0.03,0.32)
***Injecting drug use***	0.65(0.07,1.22)*	0.73(0.19,1.27)**	0.47(-0.19,1.13)	0.50(-0.11,1.12)	0.09(0.02,0.16)**	0.10(0.04,0.16)***
***Unknown***	-0.08(-1.64,1.49)	-0.22(-1.62,1.18)	0.18(-1.63,1.99)	-0.35(-1.95,1.26)	0.16(-0.06,0.37)	0.09(-0.10,0.28)
***Others***	0.06(-0.38,0.50)	0.27(-0.13,0.67)	0.11(-0.40,0.62)	0.60(0.14,1.06)*	0.05(-0.01,0.11)	0.04(-0.01,0.10)

Notes:

Model a controls for gender, ethnic, religious belief, residence, marital status, income, years of school, age, co-infection status, alcohol use, drug use, self-esteem, resilience, coping strategies, social support, depression, and anxiety

Model b controls for gender, ethnic, religious belief, residence, marital status, income, years of school, age, co-infection status, smoking, alcohol use, drug use, self-esteem, resilience, coping strategies, social support, depression, and anxiety

Model c controls for gender, ethnic, religious belief, residence, marital status, income, years of school, age, co-infection status

*p<0.05,

**p<0.01,

***p<0.001

### Subgroup analyses

In Table 3, our findings showed that among participant infected via commercial sex, perceived stigma was positively associated with having part-time (β = 1.63, 95%CI = 0.94,2.31) or full-time jobs (β = 1.42, 95%CI = 0.71,2.14), and having a higher depression score (β = 0.13, 95%CI = 0.06,0.20), while negatively associated with a higher physical activity score (β = -0.17, 95%CI = -0.28,-0.06) and having better social support (β = -0.47, 95%CI = -0.79,-0.14). Internalized stigma was positively associated with a higher coping strategy score (β = 1.14, 95%CI = 0.53, 1.75) and a higher anxiety score (β = 0.12, 95%CI = 0.07, 0.17), but negatively associated with a younger age (β = -0.04, 95%CI = -0.06,-0.01) and better social support (β = -0.80, 95%CI = -1.24,-0.35) (Table 3).

**Table 3:** Predictive model for stigma among people who infected via having sex with commercial sex partners (n = 635).

Perceived stigma (n = 635)^a^		Internalized stigma (n = 631)^b^	
Predictive Factors	(β,95%CI)	Predictive Factors	(β,95%CI)
**Age**	-0.02(-0.04,0.003)	**Age**	-0.04(-0.06,-0.01)**
**Employment**		**Attitude from medical staff-q123**	
*No work*	Ref.	*all are friendly*	ref
*Part time*	1.63(0.94,2.31)****	*majority are friendly*	-0.21(-1.05,0.63)
*Full time*	1.42(0.71,2.14)****	*half are friendly*	-1.34(-3.43,0.74)
		*less than half are friendly*	-2.31(-5.24,0.62)
**Meet the balance**		*very few are friendly*	4.85(-2.80,12.49)
*More than enough*	Ref.		
*Barely enough*	-0.12(-0.88,0.64)	**Smoking**	0.34(-0.32,0.99)
*Not enough*	0.34(-0.48,1.16)		
		**Self-rate Physical condition-q401**	
**Physical activity**	-0.17(-0.28,-0.06)**	*Excellent*	Ref.
		*very good*	0.11(-1.72,1.95)
**Self-rate Physical condition-**		*Good*	2.07(0.30,3.84)*
*Excellent*	Ref	*Average*	1.74(-0.02,3.51)
*very good*	-0.01 (-1.52,1.51)	*Poor*	1.74(-0.39,3.87)
*Good*	1.11(-0.33,2.56)		
*average*	0.52(-0.92,1.96)	**Resilience**	-0.46(-0.92,0.01)
*Poor*	0.54(-1.14,2.22)		
		**Coping strategy scores**	1.14(0.53,1.75)****
**Self-esteem**	-0.39(-0.81,0.04)		
		**Anxiety**	0.12(0.07,0.17)****
**Anxiety**	-0.03(-0.07,0.01)		
		**Social support**	-0.80(-1.24,-0.35)**
**Social support**	-0.47(-0.79,-0.14)**	**If disease affects daily life-q403**	
		*not at all*	Ref.
**Depression**	0.13 (0.06,0.20)****	*a little*	0.44(-0.49,1.37)
		*Average*	0.47(-0.60,1.53)
		*Significantly*	1.46(-0.21,3.12)
		*Very significantly*	0.83(-1.52,3.17)
		**Degree of pain-q402**	
		*not at all*	Ref.
		*very slight*	0.92(-0.17,2.00)
		*Slight*	-0.14(-1.22,0.95)
		*Average*	-0.69(-1.84,0.45)
		*Severe*	-0.27(-2.40,1.86)
		*very severe*	-1.92(-5.92,2.09)

Notes:

Model a controls for age, employment status, balance between expenditure and income, physical activity scale, self-rated physical condition, self-esteem, anxiety, social support, and depression

Model b controls for age, attitude from medical staff, smoking, self-rated physical condition, resilience, coping strategy, social support, degree to which the HIV affects daily lives, and degree of pain

*p<0.05,

**p<0.01,

****p<0.0001

In Table 4, among people who were infected via injecting drug use, perceived stigma was positively associated with part-time employment status(β = 1.03, 95%CI = 0.18, 1.89), not adhering to ART(β = 0.41, 95%CI = 0.01, 0.82), and a higher depression score(β = 0.11, 95%CI = 0.01, 0.21), but negatively associated with a higher anxiety score (β = -0.06, 95%CI = -0.11,-0.01). With multiple imputation, part time employment status (β = 0.76, 95%CI = 0.10, 1.42), a higher coping strategy score (β = 0.69, 95%CI = 0.24, 1.13), and a higher depression score (β = 0.09, 95%CI = 0.02, 0.17) were positively associated with perceived stigma; and a higher score of self-esteem (β = -0.76, 95%CI = -1.21,-0.31) was negatively associated with perceived stigma. For enacted stigma, we found the risk factors were higher years of schooling (β = 0.03, 95%CI = 0.01, 0.05), a higher physical activity score (β = 0.05, 95%CI = 0.02, 0.07), and worse anxiety score (β = 0.01, 95%CI = -0.01,0.02). Protective factors included having better self-esteem (β = -0.10, 95%CI = -0.18,-0.01) and disclosure status (β = -0.20, 95%CI = -0.34,-0.05; Table 4).

**Table 4:** Predictive model for stigma among people who infected via injecting drug use (n = 466).

	Perceived stigma^a^		Enacted stigma (n = 455)^b^	
	Complete cases^1^ (n = 283)	Multiple Imputation (n = 466)		
Predictive Factors	(β,95%CI)	(β,95%CI)	Predictive Factors	(β,95%CI)
**Attitude from medical staff**			**Years of school**	0.03(0.01,0.05)*
*all are good*	Ref.	Ref.		
*majority are good*	0.17(-0.81,1.15)	-0.70(-1.46,0.06)	**Physical activity scale**	0.05(0.02,0.07)****
*half are good*	0.33(-2.47,3.13)	0.72 (-1.00,2.43)		
*less than half are good*	0.19(-2.68,3.06)	-0.57 (-2.52,1.38)	**Alcohol use**	-0.11(-0.22,0.00)
*all are poor*	-6.06(-12.31,0.19)	-2.43(-6.70,1.85)		
			**Self-esteem**	-0.10 (-0.18,-0.01)*
**Employment status**				
*No work*	Ref.	Ref.	**If disease affects daily life**	
*Part time*	1.03(0.18,1.89)*	0.76 (0.10,1.42)*	*Not at all*	Ref.
*Full time*	0.87(-0.13,1.87)	0.73(-0.03,1.50)	*A little*	0.08(-0.07,0.23)
			*Average*	0.15(-0.02,0.32)
**No adherence**	0.41(0.01,0.82)*	0.27(-0.11,0.65)	*Significantly*	0.30(0.08,0.53)*
			*Very significantly*	0.20(-0.12,0.53)
**Self-esteem**	-0.60(-1.24,0.04)	-0.76(-1.21,-0.31)***		
			**Social support**	-0.07(-0.15,0.00)
**Resilience**	-0.38(-0.96,0.19)	-0.31(-0.75,0.12)		
			**Depression**	0.01(0.00,0.03)
**Coping strategy scores**	0.51(-0.12,1.13)	0.69(0.24,1.13)**		
			**Anxiety**	0.01(0.01,0.02)**
**Depression**	0.11(0.01,0.21)*	0.09(0.02,0.17)*		
			**Disclosure**	-0.20(-0.34,-0.05)*
**Anxiety**	-0.06(-0.11,-0.01)*	-0.04 (-0.08,0.01)		
**Disclosure**	0.68(-0.42,1.78)	0.38(-0.32,1.09)		

Notes:

Model a controls for attitudes from medical staff, employment status, no adherence, self-esteem, resilience, coping strategy, depression, anxiety and disclosure status; 1: as the percentage of missing values is over 15%, multiple imputation is employed.

Model b controls for years of schooling, physical activity score, alcohol use, self-esteem, the degree to which disease affects their daily life, social support, depression, anxiety, and disclose status

*p<0.05,

**p<0.01,

***p<0.001,

****p<0.0001

## Discussion

In the current study, we found PLWHA had high level of perceived, internalized and enacted stigma, which was consistent with findings from studies conducted in Swedish and Chinese contexts [45,46]. Our findings further confirmed our hypothesis that people who contracted HIV from “blameless” routes (e.g., with stable partners) may have less stigmatized experience compared to people who contracted HIV from “blamable” routes (e.g., injecting drug use, sex with sex workers). PLWHA who were infected via injecting illegal drugs or having sex with sex workers might be further marginalized [2,5,20,47]. A study conducted in Malaysia indicated the source of infection not only impacted the magnitude of stigma that PLWHA encountered, but also affected their quality of life and prognosis of the disease [27]. For instance, if a person is infected via sexual contact or drug use, s/he prefers not to disclose the serostatus to others; and his/her family members are less willing to provide care to them compared to their peers contracting HIV via blameless routes [27].

Our findings indicated that participants infected via injecting drug use had experienced worse physical, emotional and financial constraints compared to their peers, and they were more likely to report perceived and enacted stigma. This specific group experienced “double curses” due to their socially devalued identifies as both “HIV carriers” and “injected drug users (IDU)”. IDUs were labelled as “social evils” and have been disproportionately impacted by HIV in China[1,14]. Drug use is majorly considered as a moral weakness or deviant behaviors rather than a medical disorder[48]. Existing studies have revealed that active use of illegal drugs was a key barrier of returning to normalcy, and the main source of stigma[49].

Meanwhile, our data revealed that self-esteem, ART-adherence, disclosed serostatus, and social support might be associated with reduced stigma among the study sample. Consistent with existing literatures, self-esteem and social support not only protected them from being stigmatized, but also protected them from the negative effects of being stigmatized [50–54]. On the other hand, serostatus disclosure may exert a potential impact on reducing stigmatizing experience when appropriately conducted[55–57]. A meta-analysis of 21 studies also revealed a negative and homogenous correlation between stigma and disclosing HIV-positive status[57]. Strategies should be emphasized to increase ART adherence, social support, and cultural tolerance to facilitate communication of serostatus in future interventions among this group.

We found malfunction of mental health was a risk factor for stigma for all PLWHA regardless of infection routes. Perhaps people with better mental health status are more resilient to negative life events, or PLWAH with less stigma felt better psychologically [13,25,58]. Wu and Li (2014) have demonstrated the feasibility and positive intervention effects of community-based programs to promote psychosocial wellbeing of PLWHA in China [59]. However, there is still a scarcity of research on PLWHA’s mental health status and translation of the evidence for the development of stigma-coping strategies in China [60–63]. Thus, we urgently advocate that screening and promotion activities of mental health among PLWHA should be incorporated into existing stigma-reduction campaigns in China, as stigma reduction interventions and mental health promotion programs may work synergistically.

In our study, a relatively large proportion of participants reported “unknown” as their sources of infection. Perhaps some participants were unwilling to tell. It is also likely that a certain amount of PLWHA had multiple risk behaviors that they cannot determine which the major route of contraction was. Future research also needs to explore the specific reasons among this group as they may pose higher risks compared to their peers who knew their major routes of infection.

A few limitations should be acknowledged while interpreting findings in the current study. First, due to the cross-sectional study design, we cannot make casual inferences. A longitudinal study in future should be conducted to explore causal relationship between routes of infection and stigma. Second, because of social desirability bias, PLWHA were prone of under-reporting their illegal behaviors such as commercial sex or injecting drug use. As we could not verify the accuracy of such reporting, routes of infection may be subject to misclassification. Third, as all participants were recruited from rural areas of Guangxi province, findings in the current study may have constrained generalizability to other settings. Fourth, due to the limited space of the study survey, we did not explore if the sexual transmission was due to heterosexual or homosexual contact, which may have different risk sets from what we identified. Fifth, the current study was generated from a secondary data analysis, which may result in residual confounding due to insufficient or incomplete measurements and/or may not address the specific research question.

Despite these caveats, the large sample size, rigorous model building strategies as well as sensitivity analyses (e.g., multiple imputation) may increase the confidence of our estimations [41]. The findings shed light on stigma-reduction interventions among PLWHA in China. A better understanding of the association between routes of infection and the stigma they experienced or perceived will help health professionals and policy makers to develop tailored behavioral and policy-oriented intervention strategies for PLWHA to tackle different types of stigma in high-risk settings [64].

## Supporting Information

S1 Data Supporting Information Original Data File: *hivpos*.*dta*. (DTA)
